# Retraction: Exosome-derived PTENP1 suppresses cisplatin resistance of bladder cancer (BC) by suppressing cell proliferation, migration and inducing apoptosis *via* the miR-103a/PDCD4 axis

**DOI:** 10.1039/d1ra90073k

**Published:** 2021-02-03

**Authors:** Laura Fisher

**Affiliations:** Royal Society of Chemistry Thomas Graham House, Science Park, Milton Road Cambridge CB4 0WF UK advances-rsc@rsc.org

## Abstract

Retraction of ‘Exosome-derived PTENP1 suppresses cisplatin resistance of bladder cancer (BC) by suppressing cell proliferation, migration and inducing apoptosis *via* the miR-103a/PDCD4 axis’ by Xingre Lu *et al.*, 2019, **9**, 37642–37651, DOI: 10.1039/C9RA07823A.

The Royal Society of Chemistry hereby wholly retracts this *RSC Advances* article due to concerns with the reliability of the data. The images in the article, and raw data provided by the authors, were screened by an image integrity expert. The raw data provided by the authors was found to closely resemble raw data for a number of other articles, which is unexpected given that there are completely different authors lists for these articles.

In addition, the paper was analysed by experts who fact-checked the identities of the described nucleotide sequence reagents,^1^ and found errors with the following nucleotide sequence reagents reported in the article: miR-103a reverse primer, GAPDH forward primer, and U6 forward and reverse primers. Therefore, the results shown in Fig. 1–6 are unreliable. The reported miR-103a reverse primer is not a reverse RT-PCR primer specific for hsa-miR-103a, but a universal reverse primer. This paper therefore reports a wrongly described reagent, which impacts on the reliability of the results and may be misleading for future studies. Furthermore, the claimed identities of the nucleotide sequences reported for the GAPDH forward primer, and U6 forward and reverse primers are incorrect, and given that these reagents are used as controls, all the reported RT-PCR results are therefore unreliable.

Given the significance of the concerns about the validity of both the data in the article and the raw data provided by the authors, the findings presented in this paper are not reliable.

The authors oppose the retraction.

Signed: Laura Fisher, Executive Editor, *RSC Advances*

Date: 19^th^ January 2021

## Supplementary Material
